# Dislocation, following total knee arthroplasty

**DOI:** 10.4103/0019-5413.80051

**Published:** 2011

**Authors:** Raju Vaishya, Vikrant Landge, Shiraz Ahmad, Gaurav Neupane

**Affiliations:** *Department of Orthopedics and Joint Replacement Surgery, Indraprastha Apollo Hospitals, New Delhi, India*

Sir,

We read article on ‘Dislocation following total knee arthroplasty (TKA)’^1^ with interest. We congratulate the authors for highlighting the details of a rare complication of TKA, i.e., dislocation of the prosthesis.

Based upon our experience in dealing with six such cases of dislocation in posterior stabilized (PS) knees, we disagree with the authors regarding their method of treatment. The authors have performed a delayed revision of all components in each of their cases, without specifying the real indication for doing so. Revision TKA is a formidable surgery with relatively poor outcomes and may be associated with complications like infection etc., as happened in one of the cases in their series. We advocate that only if there is significant instability after the reduction and/or if the prosthesis components are loose and/or if the components are grossly malpositioned should the patient be subjected to revision TKA for all-component revision.^2^

**Figure 1:**
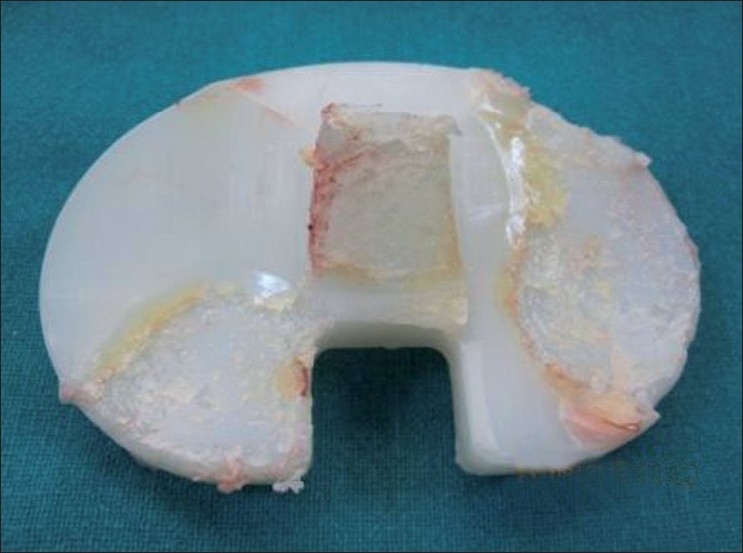
Wear on the medial side and posterior aspect of the lateral side of the polyethylene insert

Among our six cases we could achieve stable reduction in four patients with closed reduction under general anesthesia. On table, assessment under anesthesia after reduction did not reveal any gross instability and these patients were treated conservatively with a knee brace for 3 weeks post reduction. After 3 weeks, range of motion exercises of the knee was started. All patients achieved pre-dislocation status. After an average follow-up of 3.5 years there was no recurrence of instability or implant loosening. Only two patients, who had recurrent instability, required revision TKA and both of them could be salvaged with a polyethylene insert that was one size thicker. In these two cases, polyethylene wear occurred at the periphery of the tibial plateau and along the post of the tibial spine [Figure 1]; similar findings have also been reported by other surgeons.^2^

Although tibio-femoral instability has classically been reported with cruciate-retaining prostheses, we tend to agree with the authors of the article under discussion that the PS knee prosthesis is not immune to this problem^3^: it can happen in both cruciate-retaining and cruciate-substituting TKAs. A wrong surgical technique and wrong choice of constraint of the prostheses are the main causes for instability. Malalignment, malrotation, and intraoperatively uncorrected instability (especially in flexion) may lead to unstable TKA.^4^–^6^ If a minor instability due to this reason persists in a knee it may lead to early wear and/or breakage of the tibial polyethylene post (in a PS knee) and delayed stretching and rupture of the posterior cruciate ligament (PCL) in a cruciate-retaining knee, resulting in a delayed dislocation of the prosthesis, either spontaneously or after minor trauma.^7^

In the paper under discussion, the authors have recommended a universal need for revision TKA after even a single episode of dislocation. We tend to disagree and would like to point out that all patients may not require revision and some can be managed conservatively with successful outcomes.^7^ The surgeon should have a higher threshold for resorting to revision TKA in such cases. Experience from larger series and joint registries is required to enlighten us further regarding the pattern of such instabilities and the effective treatment modalities.
